# Bibliometric analysis of the Top 1000 most-cited articles in otolaryngology over the past decade: global research trends and hotspots

**DOI:** 10.3389/fsurg.2025.1552102

**Published:** 2025-02-17

**Authors:** Zhipeng Wang, Guodong Yu

**Affiliations:** 1 Department of Otolaryngology, Affiliated Hospital of Guizhou Medical University, Guiyang, China; 2 School of Clinical Medicine, Guizhou Medical University, Guiyang, China; 3 Guizhou Provincial People's Hospital Hearing Center, Guiyang, China

**Keywords:** bibliometric analysis, otolaryngology, review, discipline development, ENT

## Abstract

**Background:**

The field of otolaryngology has achieved remarkable progress over the past decade due to technological advancements and interdisciplinary integration. Understanding research trends and hotspots is essential to drive further innovation and development.

**Methods:**

A comprehensive search was conducted on Web of Science on November 22, 2024, to identify the top 1,000 most-cited otolaryngology publications from 2014 to 2024. Data were analyzed using GraphPad Prism v8.0.2, CiteSpace (6.2.4R), and VOSviewer (1.6.18) to visualize trends and research networks.

**Results:**

The annual publication volume in otolaryngology decreased after 2014, with the United States dominating in both publication count and citation frequency. Influential journals and prominent authors were identified, and research areas expanded beyond traditional clinical management to interdisciplinary fields. Chronic rhinosinusitis, olfactory dysfunction, and machine learning emerged as key research hotspots.

**Conclusion:**

Otolaryngology has made significant progress across multiple domains. Future research should focus on integrating artificial intelligence into clinical practice, fostering interdisciplinary collaborations, and advancing precision medicine and translational research. These efforts will be critical for addressing emerging challenges and capitalizing on new opportunities in the field.

## 1 Introduction

The field of otolaryngology involves multiple systems of the human body, including respiratory, digestive, and nervous systems, as well as sensory functions such as hearing, smell, and taste. In recent years, significant progress has been made due to the integration of multidisciplinary approaches and the application of new technologies. The wide variety of diseases, the rapidly developing interdisciplinary fields, and the large number of emerging studies present challenges for otolaryngology scholars to conduct in-depth research and explore frontier directions. Therefore, it is necessary to provide an overall overview of the current development of global research in otolaryngology.

In the past 10 years, the scope of otolaryngology research has greatly expanded, with many major advancements. Technological innovation and revolutions have provided us with valuable tools, and research into molecular and genetic mechanisms has deepened our understanding of diseases. Increasing numbers of non-otolaryngologists have joined the research on otolaryngological diseases, covering a wide range of topics from the genetic basis of hearing loss to the role of immunotherapy in head and neck cancers, the evolution of minimally invasive surgical techniques, and the application of artificial intelligence in otolaryngology. Research in otolaryngology has not only expanded our understanding of disease mechanisms but has also directly impacted clinical practice, improving patient survival quality and prognosis through more precise diagnosis, personalized treatment, and innovative surgical techniques.

This paper aims to analyze the 1,000 most cited papers in the field of otolaryngology over the past decade. By studying and analyzing these publications, our goal is to summarize the major achievements in otolaryngology research, with a focus on the progress in genetic and molecular research, advancements in diagnostic technologies, innovative treatment strategies, and the evolving surgical practices over the past 10 years, from their inception to their complex development. Additionally, we will explore future directions for otolaryngology research, considering emerging trends such as artificial intelligence, regenerative medicine, and the integration of multidisciplinary approaches into clinical practice. We will examine how these trends have emerged, in what form, and how they might impact the future of otolaryngology.

In this process, this paper will use big data processing methods and artificial intelligence tools, standing at a new height to retrospectively review the past 10 years of otolaryngology. We aim to provide a comprehensive understanding of the developmental trajectory of otolaryngology over the past decade, giving us a general impression and helping us understand what the predecessors of otolaryngology have accomplished, what footprints they have left behind, and where we can continue to break through in research.

## 2 Methods and materials

2.1 We conducted a search on Web of Science (WOS) on November 22, 2024, for all publications classified under the otolaryngology field from 2014 to November 10, 2024, with the search formula: PY = (2014–2024)

**Figure 1:**
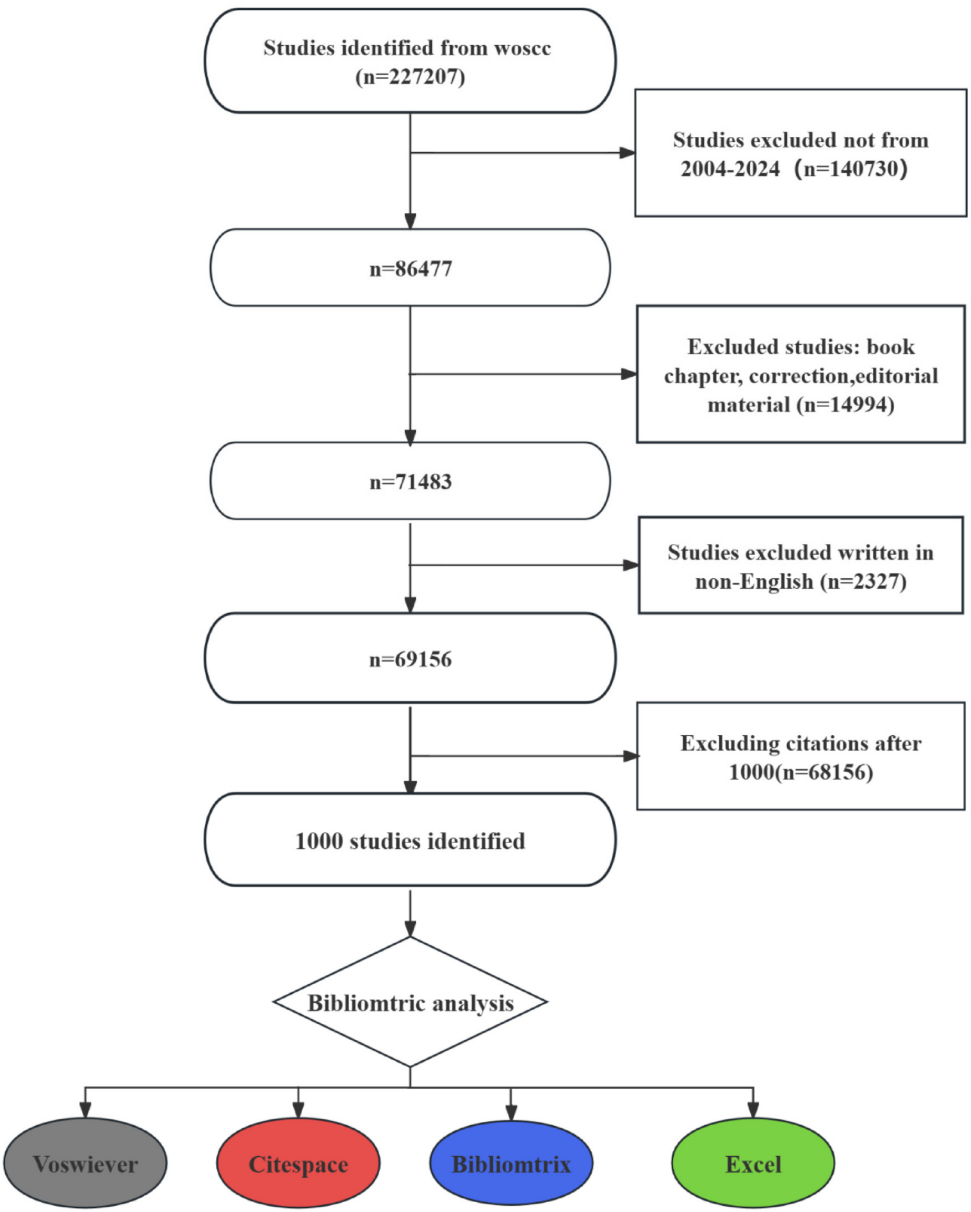
Flowchart of literature search.

AND WC = (“Otorhinolaryngology”). The top 1,000 most cited papers were selected for analysis. The inclusion criteria for the literature screening were as follows: (1) the publication is classified under otolaryngology-related disciplines; (2) the article and review manuscripts are written in English. The exclusion criteria were: (1) topics unrelated to otolaryngology; (2) conference abstracts, news articles, briefings, etc. (Figure 1). We exported the full-text versions and abstracts of the 1,000 papers in plain text format.

2.2 The obtained literature was analyzed using GraphPad Prism v8.0.2, CiteSpace [6.2.4R (64 bit)], and VOSviewer (1.6.18), and the data were visualized based on bibliometric principles.

## 3 Results

The results show that among the 1,000 most cited papers in otolaryngology over the past 10 years, there are 712 research articles and 288 review papers. The literature covers 60 countries and regions, 1,547 institutions, and 4,654 authors.

### 3.1 Annual trends

**Figure 2:**
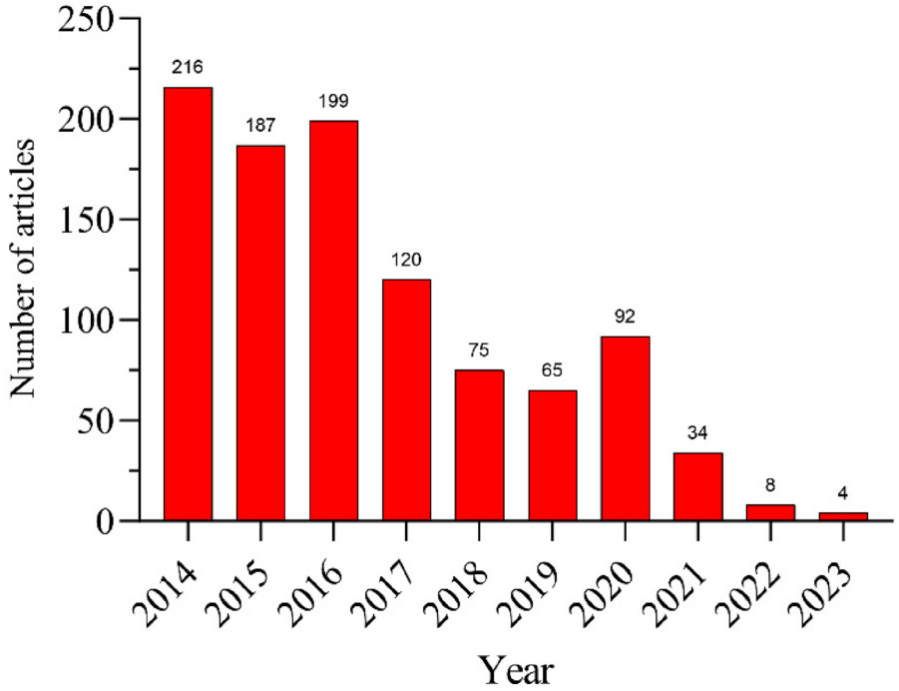
Annual volume of publications.

Since 2014, the number of papers published each year has shown a slow declining trend. The publication numbers for 2014, 2016, and 2015 were the top three years for article output. After these years, the number of highly cited publications gradually decreased year by year. By 2023, there were only 4 papers published, and no papers from 2024 have yet entered our ranking. This could be because recently published papers have fewer opportunities for citation compared to earlier studies (Figure 2).

### 3.2 Countries, regions, and institutions

**Figure 3:**
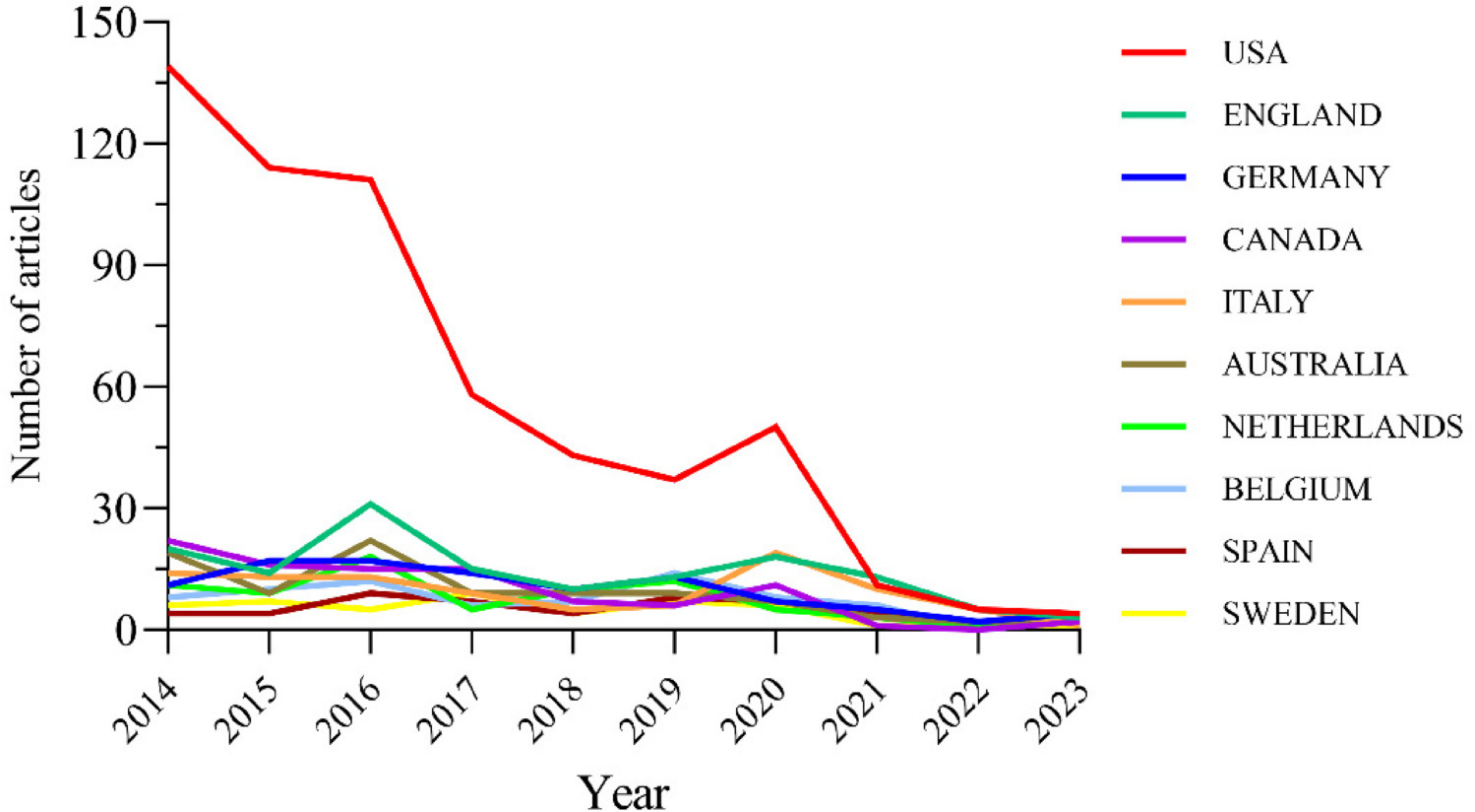
Line graph of national publications.

**Figure 4:**
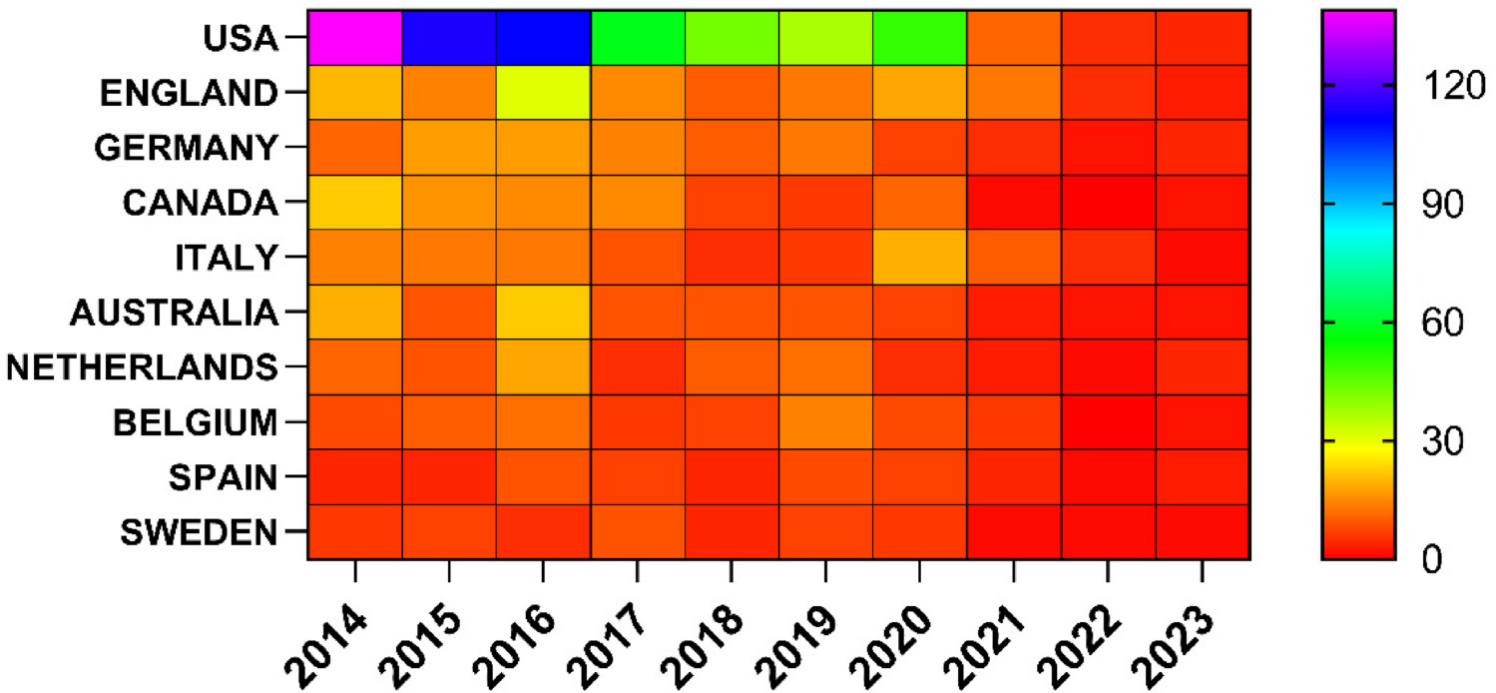
Heat map of national publications.

The 1,000 published papers come from institutions across 60 countries and regions. Figures 3, 4 show the annual publication volume of the top 10 countries over the past decade, ranking the countries by the number of papers published. The top 5 countries are the United States, the United Kingdom, Germany, Canada, and Italy. The number of papers published by the United States accounts for 57.2% of the total publications, which is four times higher than the second-ranked country and far exceeds the total number of papers from all other countries combined.

**Figure 5:**
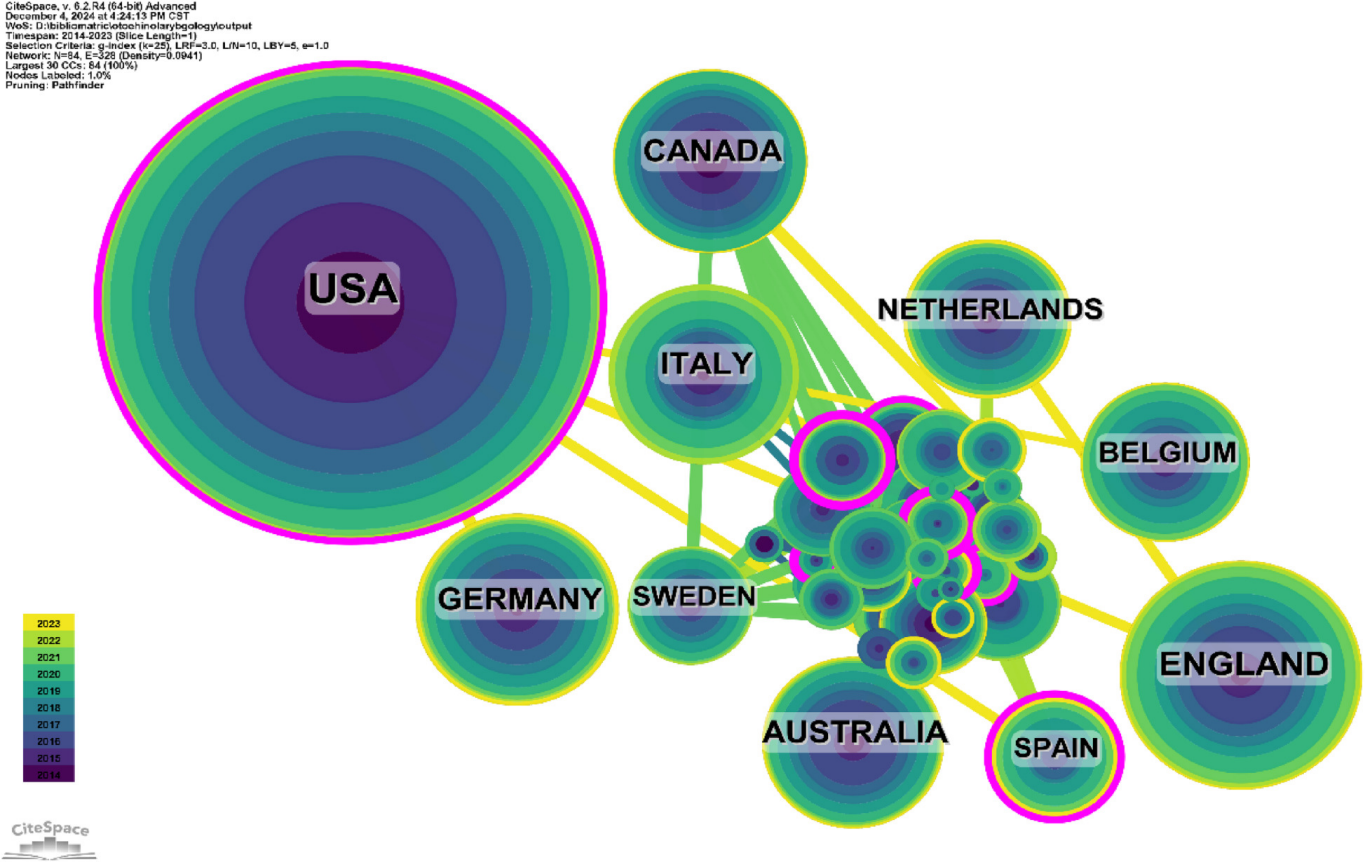
Networks of country cooperation.

Among the top ten countries and regions in terms of publication volume, the United States' papers were cited 73,153 times (Table 1), far surpassing all other countries/regions and nearly five times more than the second-ranked country. Its citation-to-publication ratio (127.89) ranks 8th among all countries. The United Kingdom, with 142 publications, ranks second in terms of publication volume, with 18,348 citations, and its citation-to-publication ratio (129.21) ranks 7th. The collaboration network is shown in Figure 5. The size of the nodes reflects the research output of the country in relevant literature, while different colors represent the output from different years. The straight lines between nodes represent collaborations between countries, with thicker lines indicating more frequent collaborations, and the color of the lines representing collaborations from different years.The United Kingdom collaborates closely with the United States, while the United States has stronger collaborations with countries like Italy, Germany, and Canada. Not only does the United States have a large number of publications and high citation frequency, but it also has a centrality score of 0.11, indicating that it is the most influential country in the field of otolaryngology. In addition, countries and regions with relatively high centrality include Spain, Canada, Italy, the Netherlands, and Sweden. In contrast, although the United Kingdom, Germany, Australia, and Belgium have citation counts exceeding 10,000, their centrality scores are relatively low, suggesting that their research tends to focus more on domestic collaborations rather than international cooperation. The top three countries in terms of citation-to-publication ratio are Spain, Canada, and Sweden, indicating that otolaryngology research conducted in these countries is more likely to be cited and of higher research quality.

**Table 1:** Table of country published literature.

Rank	Country/region	Article counts	Centrality	Percentage (%)	Citation	Citation per publication
1	USA	572	0.11	57.2%	73,153	127.89
2	ENGLAND	142	0	14.2%	18,348	129.21
3	GERMANY	100	0	10.0%	13,897	138.97
4	CANADA	95	0.07	9.5%	16,371	172.33
5	ITALY	95	0.05	9.5%	14,324	150.78
6	AUSTRALIA	91	0	9.1%	10,672	117.27
7	NETHERLANDS	78	0.03	7.8%	9,632	123.49
8	BELGIUM	73	0	7.3%	10,676	146.25
9	SPAIN	51	0.15	5.1%	8,997	176.41
10	SWEDEN	47	0.01	4.7%	7,159	152.32

**Figure 6:**
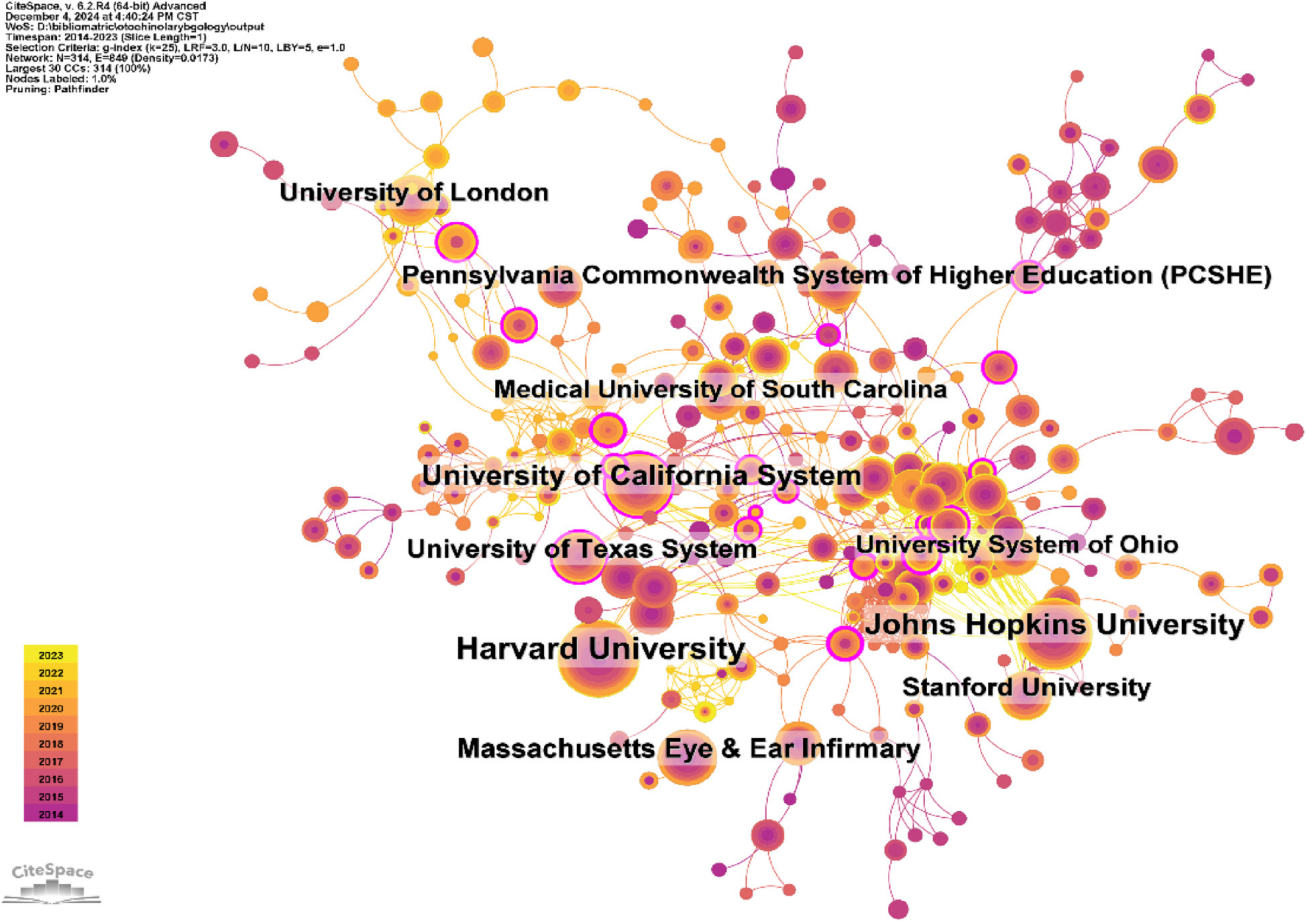
Networks of institutional co-operation.

A total of 1,547 institutions published the 1,000 otolaryngology-related articles. Among the top ten institutions in terms of publication volume, 9 are from the United States, and 1 is from the United Kingdom (Table 2, Figure 6). Harvard University published the most papers (91 papers, 13,467 citations, 147.99 citations per paper). Johns Hopkins University ranked second (75 papers, 11,250 citations, 150.00 citations per paper), followed by the University of California System (60 papers, 9,249 citations, 154.15 citations per paper) in third place. Massachusetts Eye & Ear Infirmary ranked fourth (46 papers, 6,335 citations, 137.72 citations per paper). The Medical University of South Carolina ranked ninth in terms of publication volume, but it achieved the highest average citation score (201.41). Other institutions with relatively high average citation scores include the University of London and the University of Texas System, which are ranked second and third, respectively.

**Table 2:** Table of institutional published literature.

Rank	Institution	Country	Number of studies	Total citations	Average citation
1	Harvard University	USA	91	13,467	147.99
2	Johns Hopkins University	USA	75	11,250	150.00
3	University of California System	USA	60	9,249	154.15
4	Massachusetts Eye & Ear Infirmary	USA	46	6,335	137.72
5	University of London	England	43	7,887	183.42
6	Stanford University	USA	42	5,918	140.90
7	University of Texas System	USA	42	6,880	163.81
8	Pennsylvania Commonwealth System of Higher Education (PCSHE)	USA	41	5,638	137.51
9	Medical University of South Carolina	USA	37	7,452	201.41
10	University System of Ohio	USA	36	4,173	115.92

### 3.3 Journals

**Figure 7:**
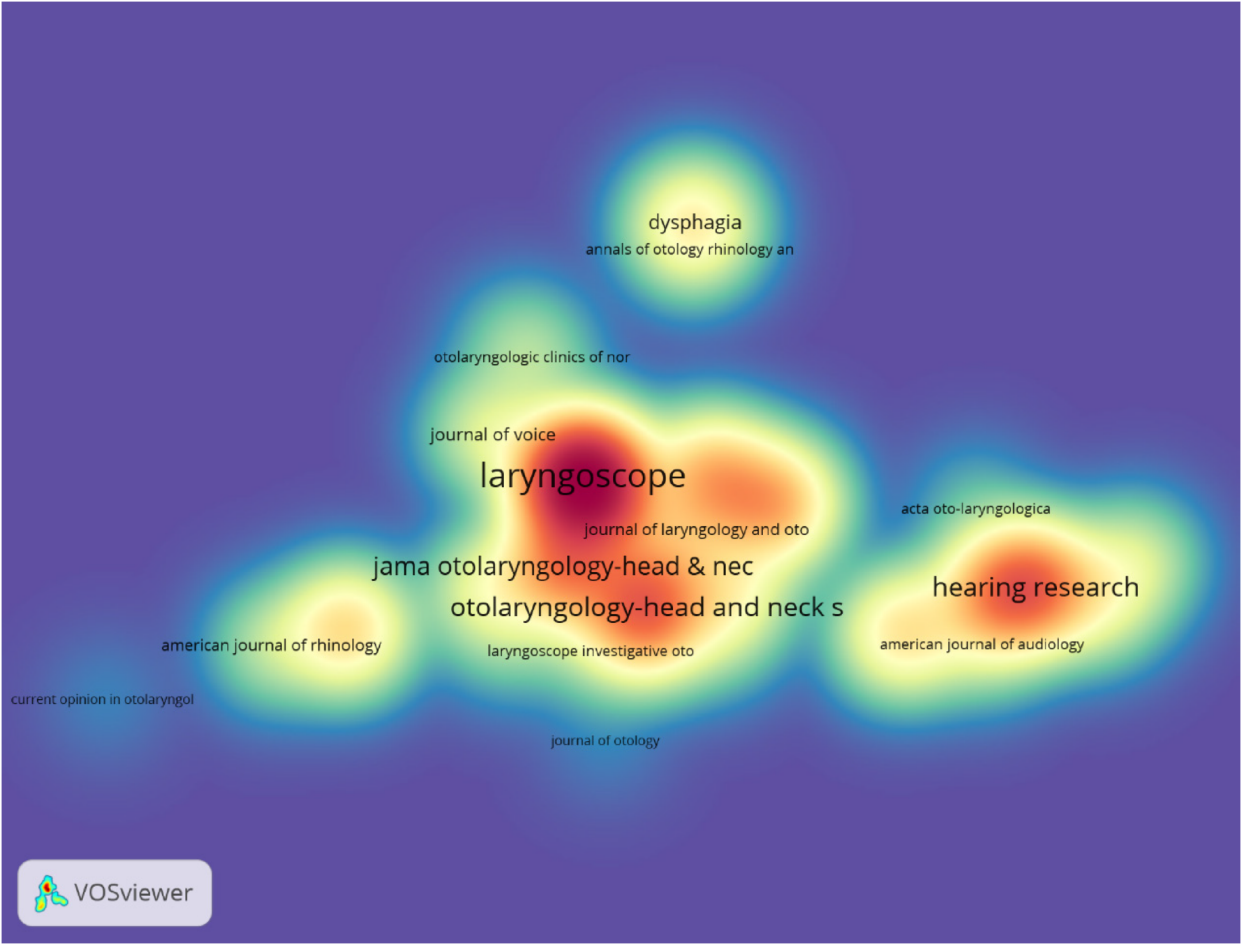
Density map of journal publications.

Table 3 and Figure 7 list the top 10 journals with the highest publication output. In the density map of journal publications, regions with different colors represent distinct research topics or clusters. The color gradient ranges from blue to red, with red areas indicating higher research activity or a larger number of related publications, while blue areas are relatively lower. The distance between journals reflects the degree of collaboration between them. *Laryngoscope* (143 papers, 14.30%) is the journal with the most publications in this field, followed by *Head and Neck—Journal for the Sciences and Specialties of the Head and Neck* (99 papers, 9.90%), *Hearing Research* (83 papers, 8.30%), and *Otolaryngology-Head and Neck Surgery* (82 papers, 8.20%). Among the top 10 most productive journals, *International Forum of Allergy & Rhinology* has the highest impact factor (IF) of 7.2. All the journals are classified in the Q1/Q2 quartiles.

**Table 3:** Table of journal publications.

Rank	Journal	Article counts	Percentage (1,000)	IF	Quartile in category
1	laryngoscope	143	14.30%	2.2	Q1
2	Head and neck-journal for the sciences and specialties of the head and neck	99	9.90%	2.4	Q2
3	Hearing research	83	8.30%	2.5	Q1
4	Otolaryngology-head and neck surgery	82	8.20%	2.7	Q1
5	Jama otolaryngology-head&neck surgery	75	7.50%	6.1	Q1
6	Otology & neurotology	58	5.80%	1.9	Q2
7	European archives of oto-rhino-laryngology	50	5.00%	1.9	Q1
8	Ear and hearing	48	4.80%	2.6	Q1
9	International forum of allergy & rhinology	45	4.50%	7.2	Q1
10	Dysphagia	34	3.40%	2.2	Q1

**Figure 8:**
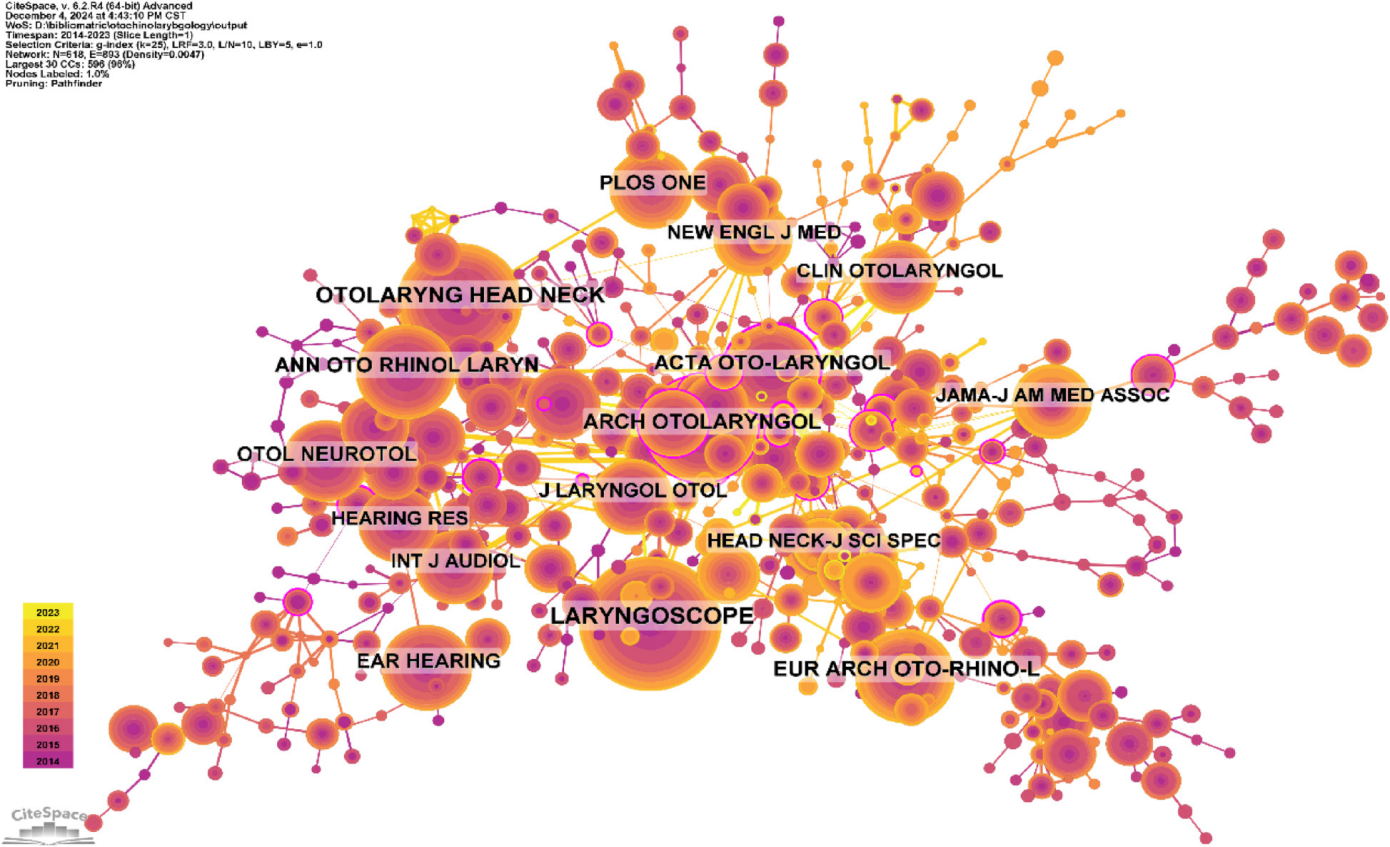
Co-citation network map of journals.

Table 4 and Figure 8 list the top ten most cited journals. The journal with the most co-citations is Laryngoscope (666 times), followed by Otolaryngology-Head and Neck Surgery (518 times) and Archives of Otolaryngology (399 times). Among the top 10 most co-cited journals, Otolaryngology-Head and Neck Surgery was cited 518 times and has the highest impact factor (IF) of 2.7. Among the co-cited journals, 70% are in the Q1/Q2 quartiles.

**Table 4:** Co-citation table of journals.

Rank	Cited Journal	Co-Citation	IF (2023)	Quartile in category
1	LARYNGOSCOPE	666	2.2	Q1
2	OTOLARYNG HEAD NECK	518	2.7	Q1
3	ARCH OTOLARYNGOL	399	–	–
4	EUR ARCH OTO-RHINO-L	346	1.9	Q2
5	ANN OTO RHINOL LARYN	338	1.3	Q3
6	ACTA OTO-LARYNGOL	311	1.3	Q3
7	EAR HEARING	279	2.6	Q1
8	OTOL NEUROTOL	255	1.9	Q2
9	PLOS ONE	251	2.7	Q1
10	HEAD NECK-J SCI SPEC	220	2.4	Q1

### 3.4 Topic distribution

**Figure 9:**
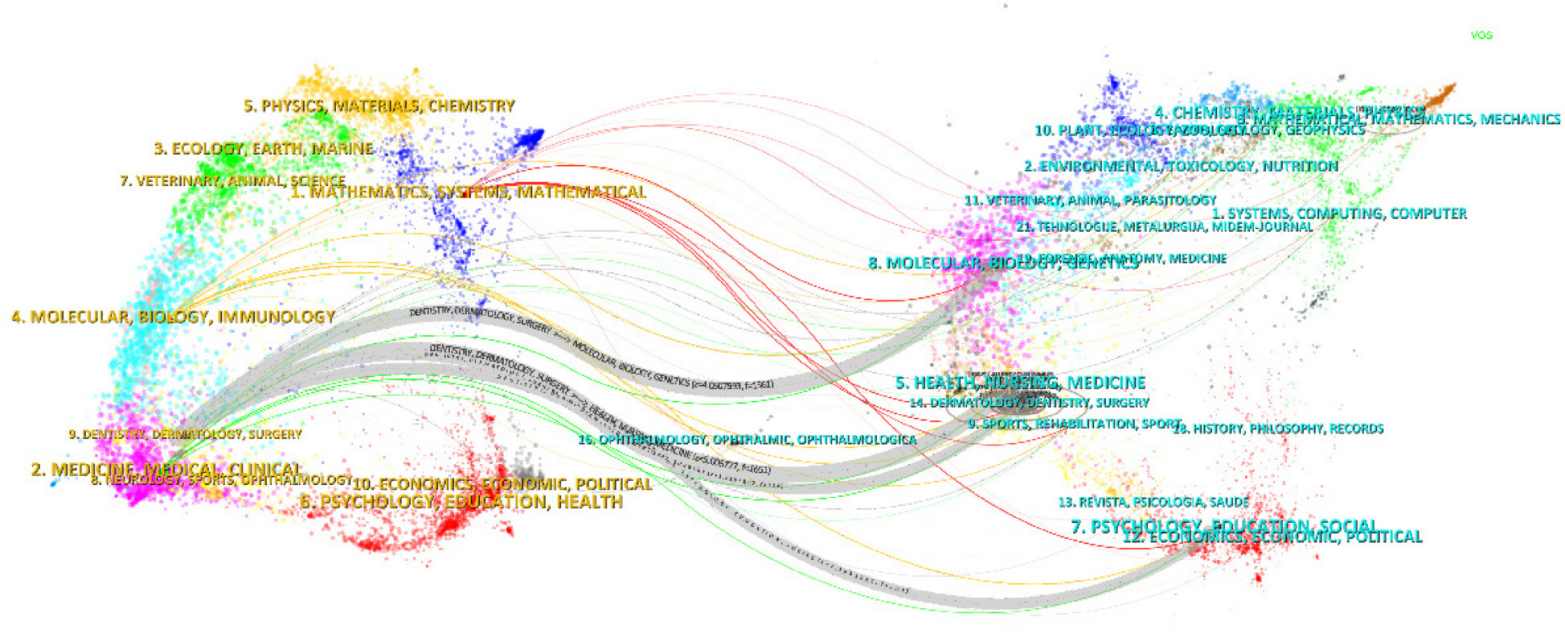
Dual map of journals.

The topic distribution of academic publications is displayed through a double-map overlay (Figure 9). The colored trajectories in the double-map overlay represent citation relationships, with citing journals on the left and cited journals on the right. Based on the displayed results, we identified two main colored citation paths. Research published in journals in the fields of health/nursing/medicine, psychology/education/social, dermatology/dentistry/surgery, and molecular/biology/genetics are primarily cited by research published in journals in the fields of dentistry/dermatology/surgery.

### 3.5 Authors and co-cited authors

**Figure 10:**
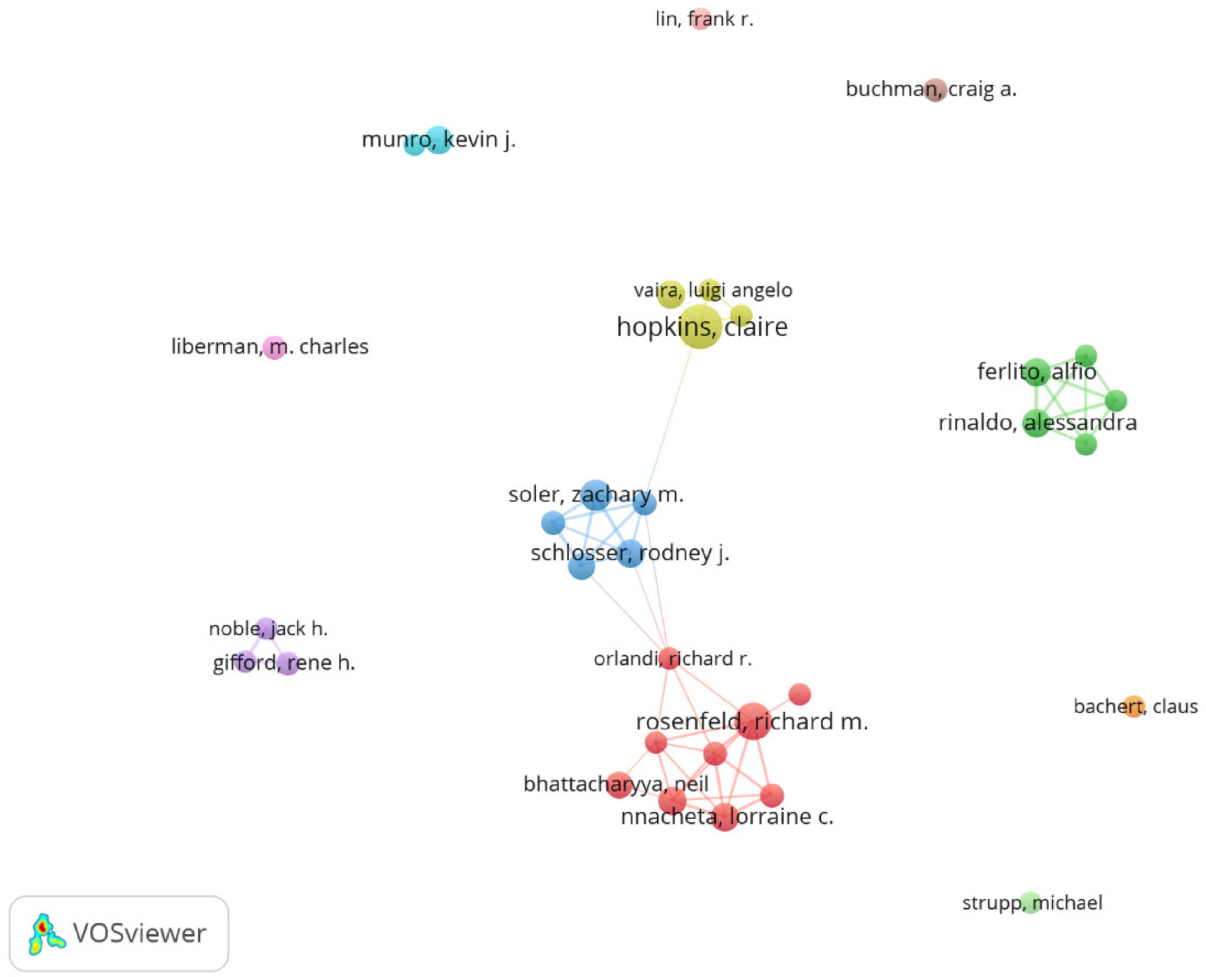
Cooperation network of authors.

Among all the authors who published the 1,000 articles, Table 5 lists the top 10 authors with the most publications. The top 10 authors collectively published 122 papers, accounting for 12.2% of all papers in the field. These authors are significant researchers in the field of otolaryngology. Hopkins, Claire, has the most published papers, with 18 articles, followed by Rosenfeld, Richard M. (15 papers) and Soler, Zachary M. (12 papers). CiteSpace visualizes the network of authors (Figure 10).

**Table 5:** Author's publications and co-citation table.

Rank	Author	Count	Rank	Co-cited author	Citation
1	Hopkins, claire	18	1	Bhattacharyya n	53
2	Rosenfeld, richard m.	15	2	Hopkins c	51
3	Soler, zachary m.	12	3	Kujawa sg	46
4	Ferlito, alfio	11	4	Rosenfeld rm	46
5	Hummel, thomas	11	5	Lin fr	44
6	Munro, kevin j.	11	6	Liberman mc	42
7	Nnacheta, lorraine c.	11	7	Liberati a	42
8	Rinaldo, alessandra	11	8	Lechien jr	40
9	Schlosser, rodney j.	11	9	Fokkens wj	38
10	Schwartz, seth r.	11	10	Soler zm	38

**Figure 11:**
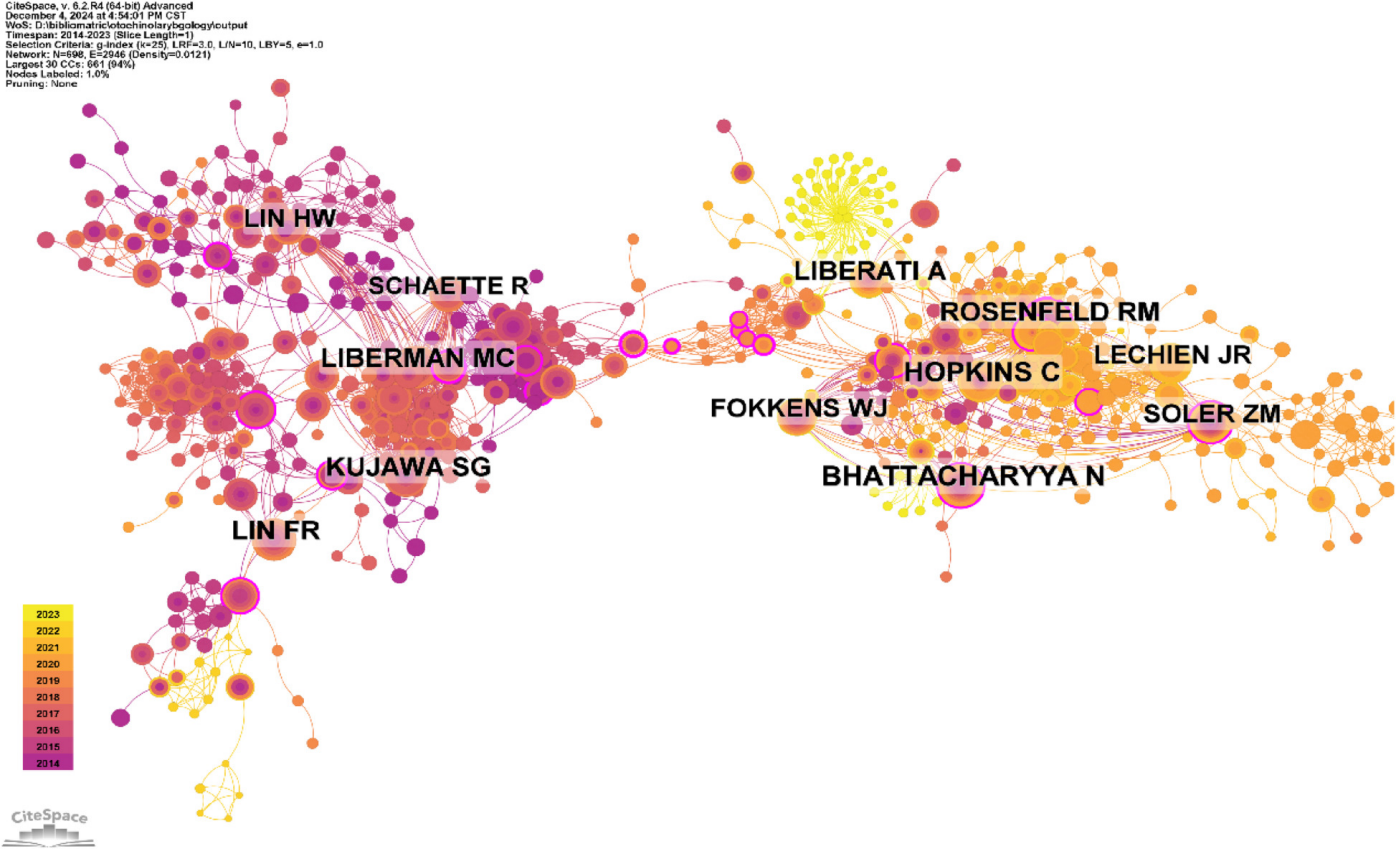
Co-citation network of authors.

Figures 11 and Table 5 show the top 10 authors with the most co-citations and the highest citation counts. These 15 authors were cited more than 444 times in total, indicating that their research has high reputation and influence. The largest nodes are related to the most co-cited authors, including Bhattacharyya N (53 citations), Hopkins C (51 citations), and Kujawa SG (46citations).

### 3.6 Co-cited references

**Figure 12:**
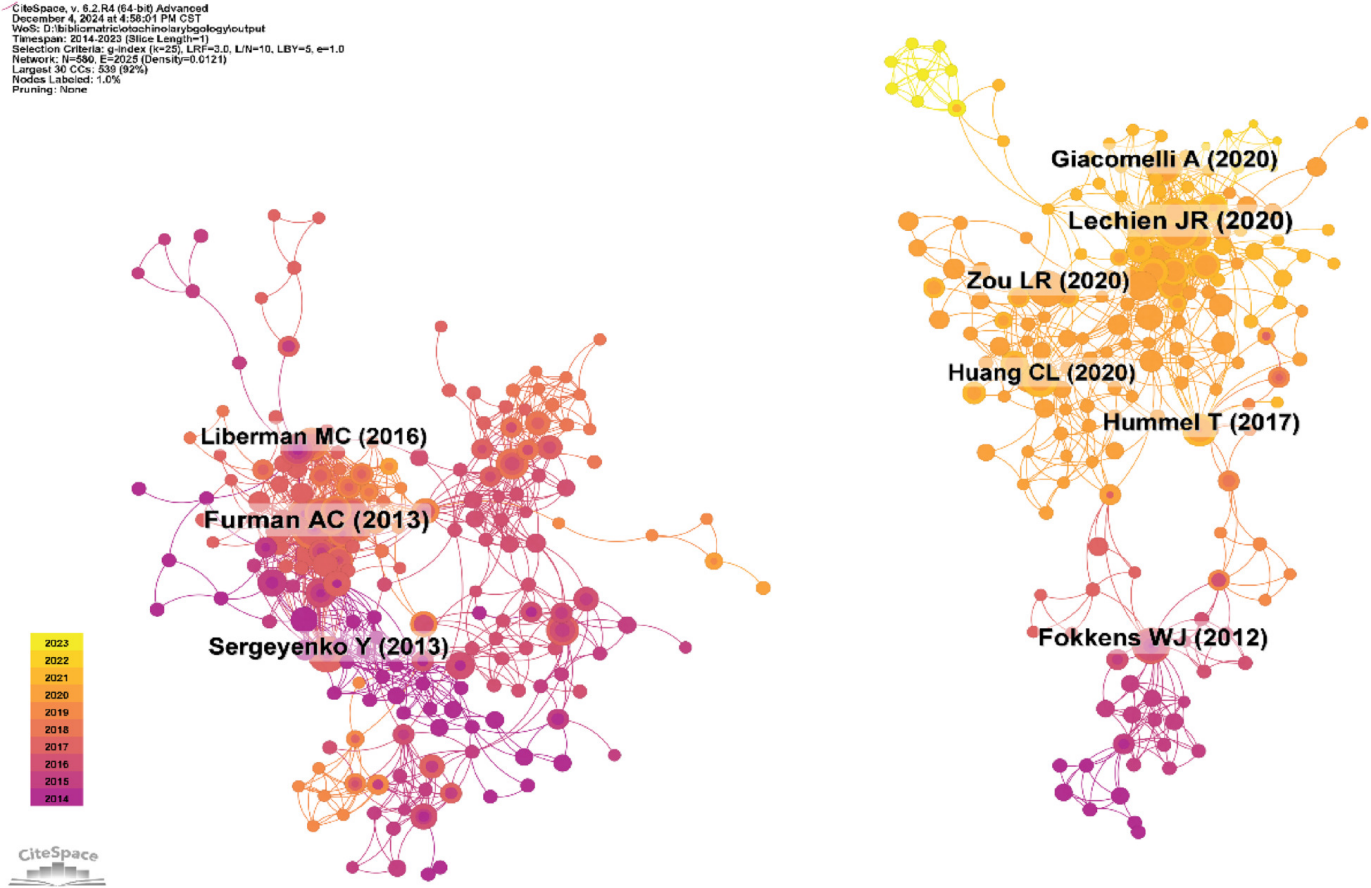
Co-cited network of literature.

Using a time slice of one year, with a time range from 2014 to 2023, the co-cited reference network consists of 580 nodes and 2025 links (Figure 12). Based on the top 10 most co-cited articles (Table 6).

**Table 6:** Co-citation table of literature.

Rank	Title	Journal	author(s)
1	Noise-induced cochlear neuropathy is selective for fibers with low spontaneous rates	JOURNAL OF NEUROPHYSIOLOGY	Stead LF
2	Olfactory and gustatory dysfunctions as a clinical presentation of mild-to-moderate forms of the coronavirus disease (COVID-19): a multicenter European study	EUROPEAN ARCHIVES OF OTO-RHINO-LARYNGOLOGY	Jorenby DE
3	Age-Related Cochlear Synaptopathy: An Early-Onset Contributor to Auditory Functional Decline	JOURNAL OF NEUROSCIENCE	Gonzales D
4	SARS-CoV-2 Viral Load in Upper Respiratory Specimens of Infected Patients	NEW ENGLAND JOURNAL OF MEDICINE	Hughes JR
5	Position paper on olfactory dysfunction	AM J ADDICTION	Kosten TR
6	Self-reported Olfactory and Taste Disorders in Patients With Severe Acute Respiratory Coronavirus 2 Infection: A Cross-sectional Study	LANCET PSYCHIAT	Horowitz MA
7	Toward a Differential Diagnosis of Hidden Hearing Loss in Humans	ADDICT BEHAV	Davies J
8	Clinical features of patients infected with 2019 novel coronavirus in Wuhan, China	PSYCHOPHARMACOLOGY	Roberts V
9	Factors Affecting Open-Set Word Recognition in Adults With Cochlear Implants	NICOTINE TOB RES	Fagerstrom K
10	Individual Differences Reveal Correlates of Hidden Hearing Deficits	NICOTINE TOB RES	Shiffman S

### 3.7 Co-cited references clustering and time cluster analysis

**Figure 13:**
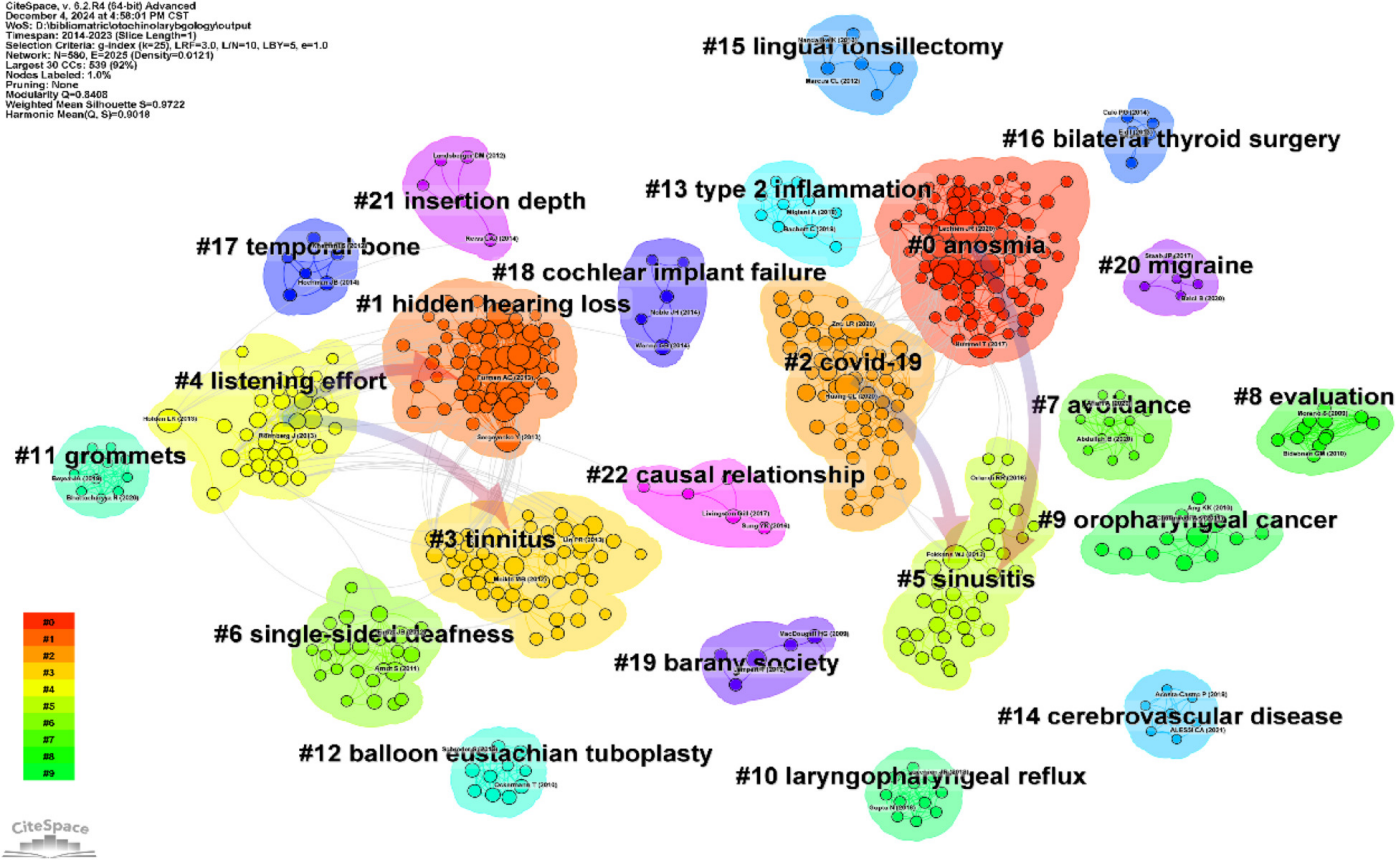
Clustering of co-cited literature.

**Figure 14:**
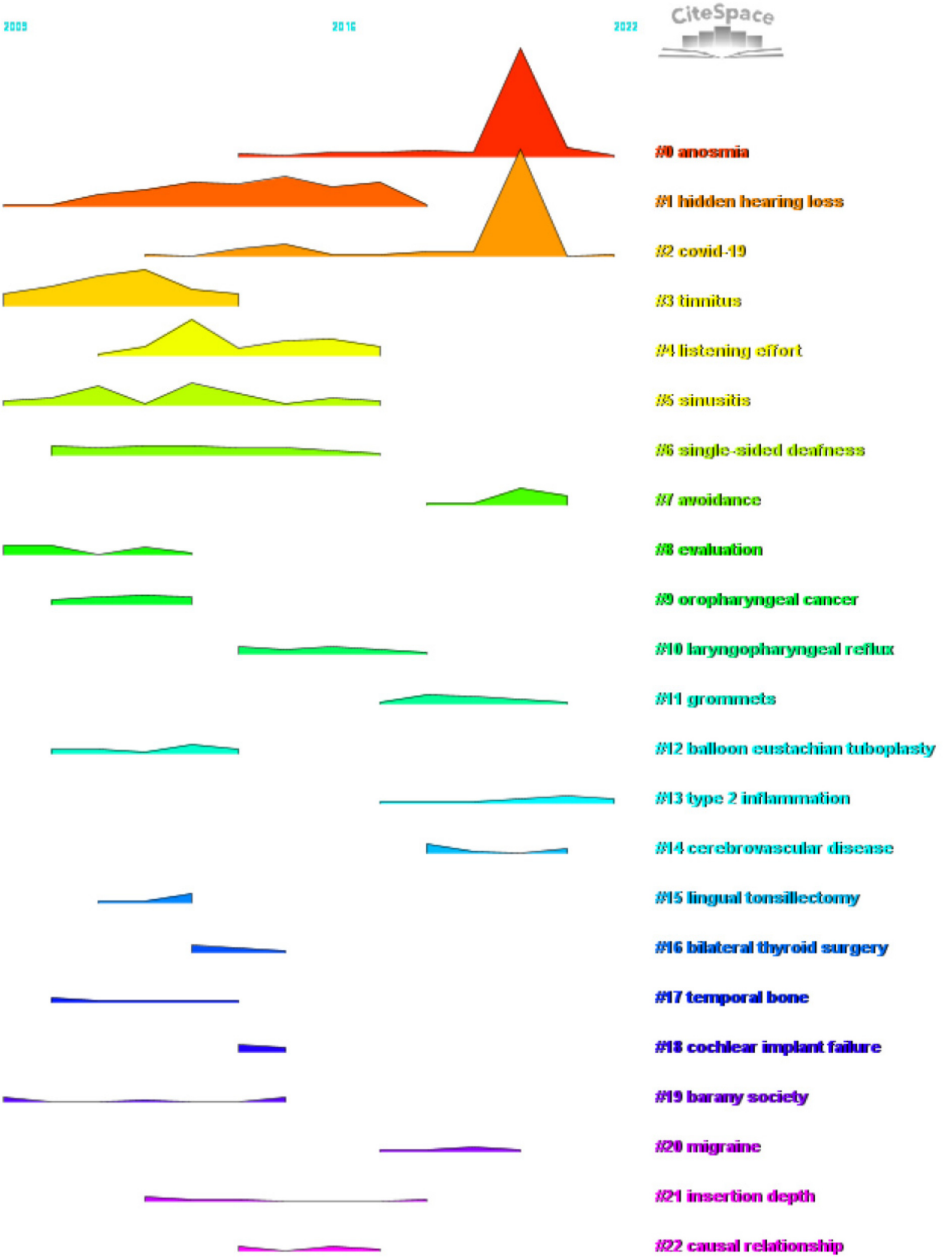
Peak map of co-cited literature.

We conducted co-cited reference clustering and time cluster analysis (Figures 13, 14). We found that early research hotspots include *tinnitus* (Cluster 3), *evaluation* (Cluster 8), *oropharyngeal cancer* (Cluster 9), *balloon Eustachian tuboplasty* (Cluster 12), *lingual tonsillectomy* (Cluster 15), *temporal bone* (Cluster 17), and the *Barany Society* (Cluster 19). Mid-term research hotspots include *hidden hearing loss* (Cluster 1), *listening effort* (Cluster 4), *sinusitis* (Cluster 5), *single-sided cleftness* (Cluster 6), *laryngopharyngeal reflux* (Cluster 10), *bilateral thyroid surgery* (Cluster 16), *cochlear implant failure* (Cluster 18), *insertion depth* (Cluster 21), and *causal relationship* (Cluster 22). Current hot topics and trends in the field include *anosmia* (Cluster 0), *COVID-19* (Cluster 2), *avoidance* (Cluster 7), *grommets* (Cluster 11), *type 2 inflammation* (Cluster 13), *cerebrovascular disease* (Cluster 14), and *migraine* (Cluster 20).

### 3.8 Keyword analysis

**Figure 15:**
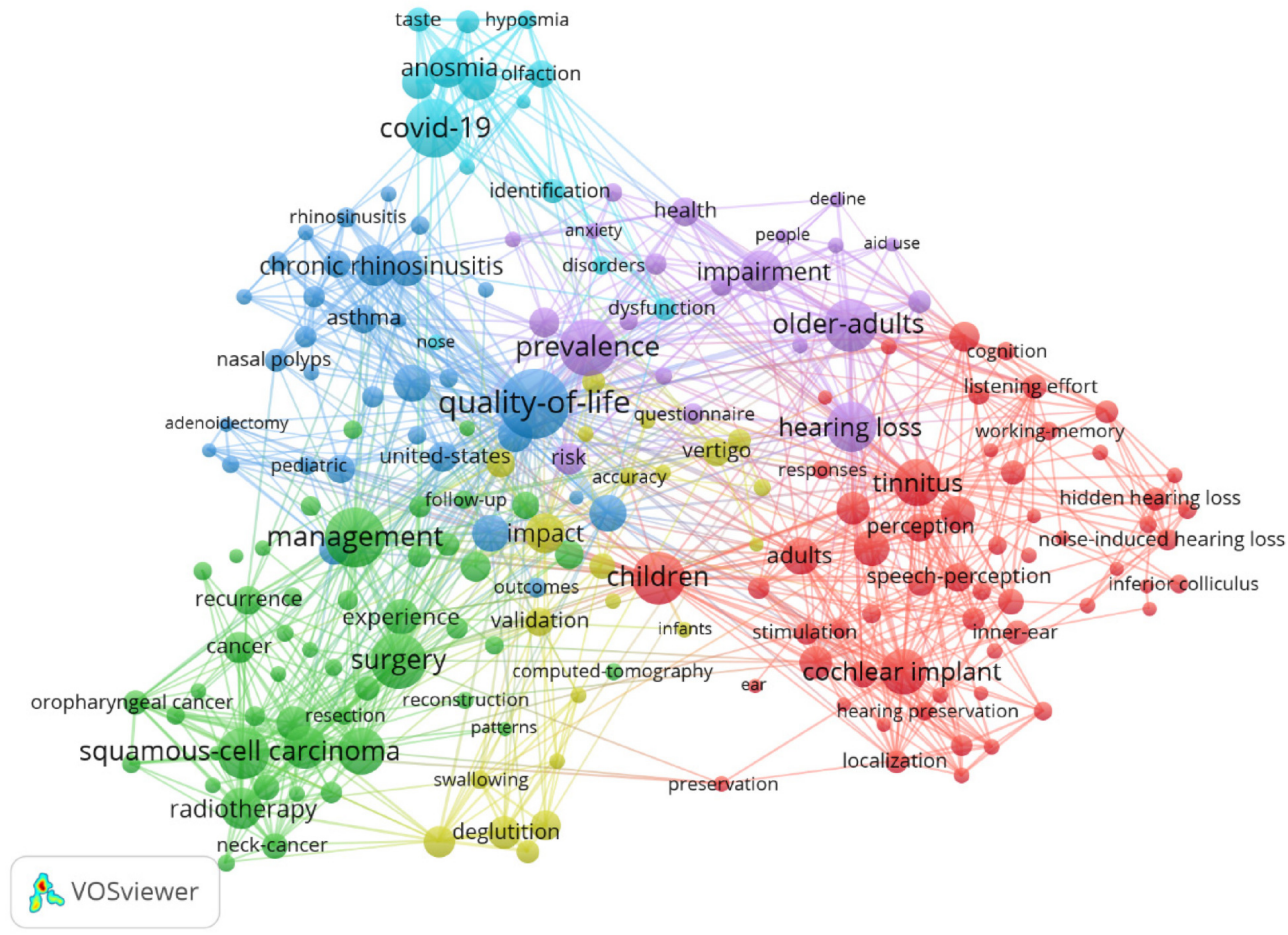
Network map of high-frequency keywords.

**Figure 16:**
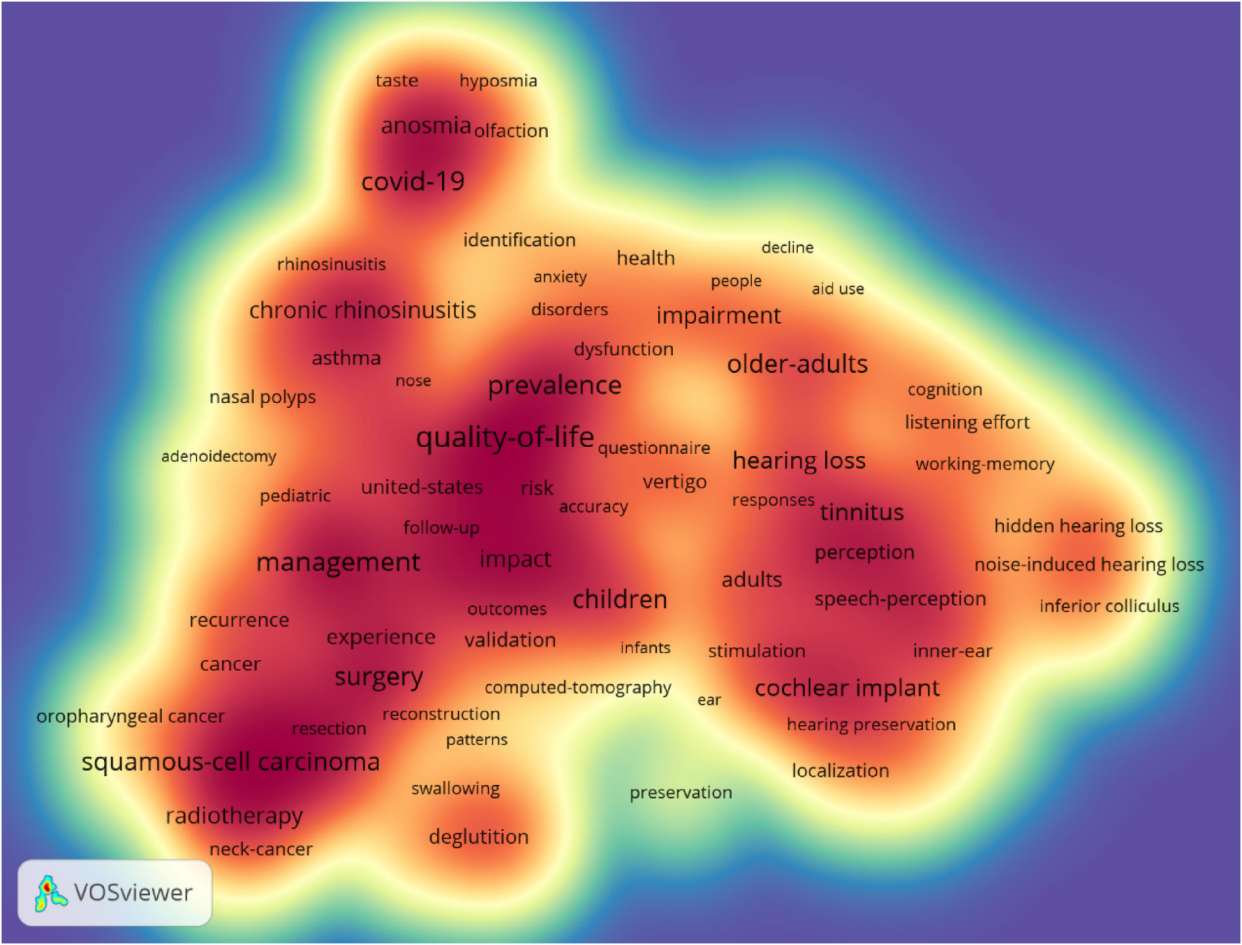
Density map of keywords.

**Figure 17:**
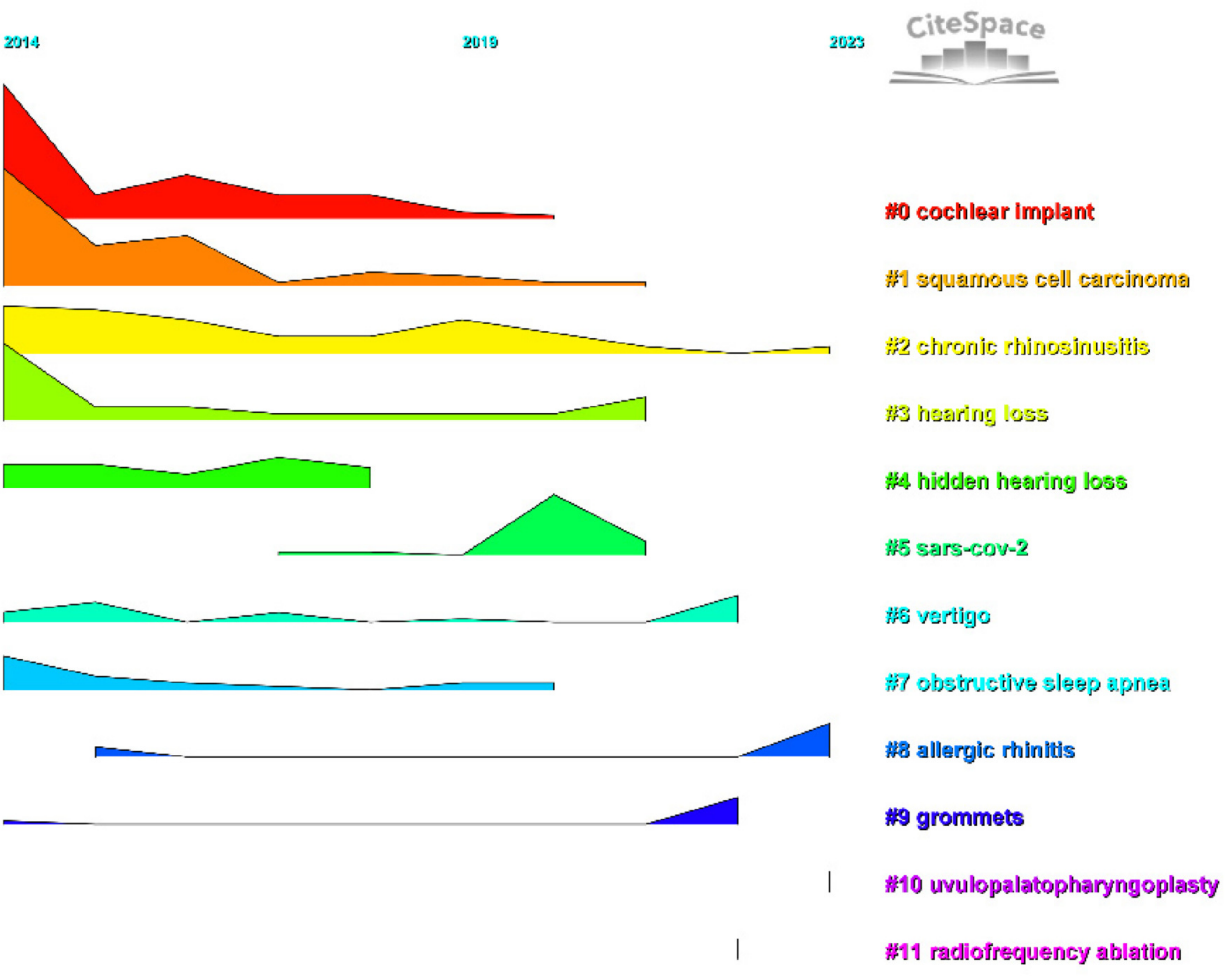
Peak map of keyword clustering.

**Figure 18:**
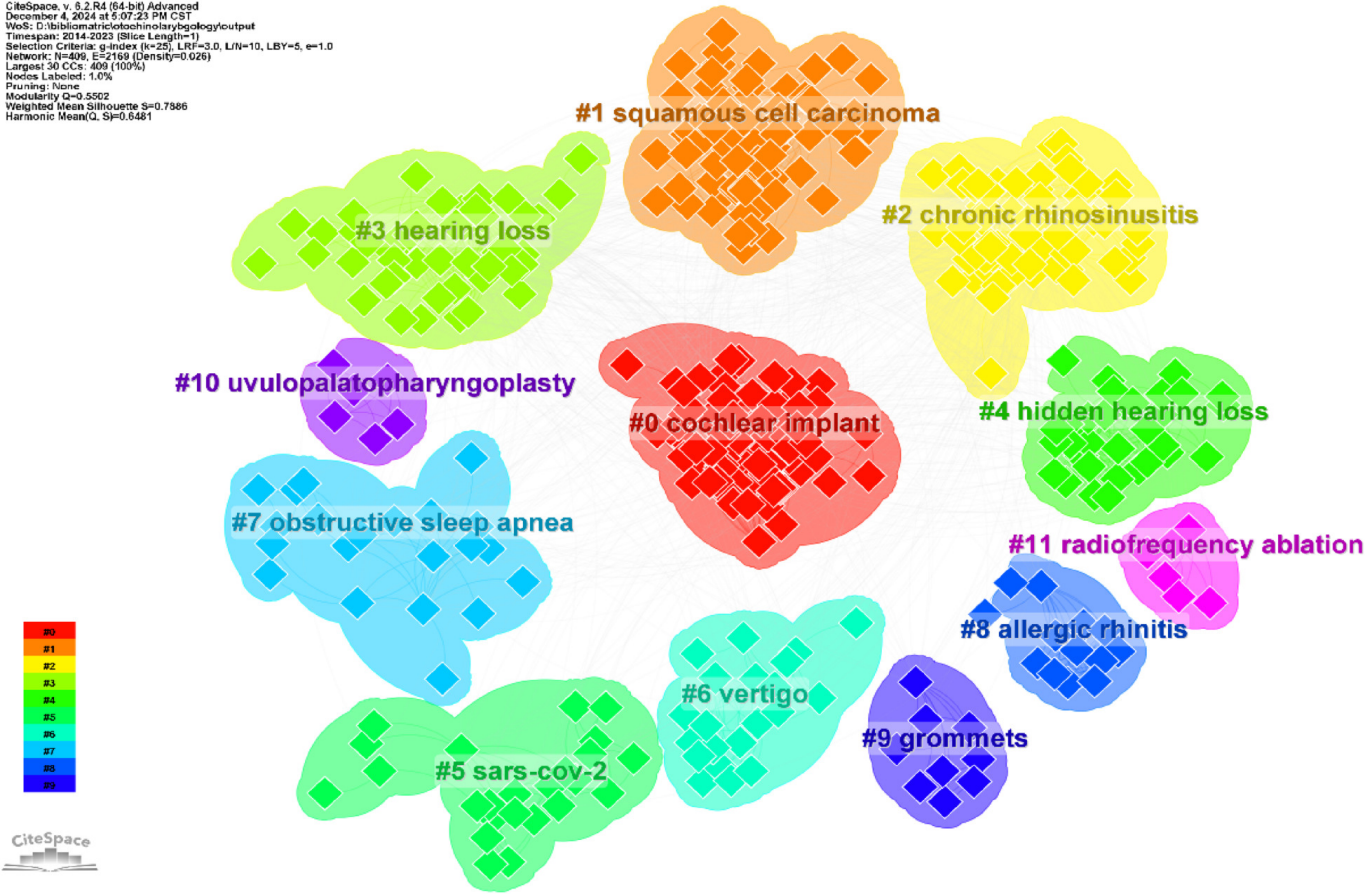
Clustering map of keywords.

By analyzing keywords, we can quickly understand the state and development direction of a field. Based on the co-occurrence of keywords in VOSviewer, the most popular keywords are *quality of life* (91), followed by *COVID-19* (71), *prevalence* (68), and *surgery* (66) (Table 7, Figures 15, 16). We removed irrelevant keywords and constructed a network containing 192 keywords that appeared at least 9 times, resulting in 6 distinct clusters. Cluster 1 (Red) contains 59 keywords, including *tinnitus*, *children*, *cochlear implant*, *brain stem*, *hidden hearing loss*, *noise*, *aging*, *attention*, *cochlear implants*, *fatigue*, *histopathology*, *electrical stimulation*, *individual difference*, *inferior colliculus*, *inner ear*, *recognition*, *residual hearing*, *sensitivity*, *speech*, and *unilateral deafness*. Cluster 2 (Green) contains 48 keywords, including *management*, *survival*, *head and neck cancer*, *radiotherapy*, *surgery*, *recurrence*, *chemotherapy*, *endoscopy*, *follow-up*, *inflammation*, *meta-analysis*, *paranasal sinuses*, *pattern*, *prognosis*, *radiation*, *reconstruction*, *risk factor*, *tracheostomy*, and *trial*. Cluster 3 (Blue) contains 33 keywords, including *quality of life*, *epidemiology*, *asthma*, *chronic rhinosinusitis*, *adenoidectomy*, *allergy*, *diagnosis*, *efficacy*, *model*, *obstructive sleep apnea*, *pediatrics*, *sleep*, and *treatment*. Cluster 4 (Yellow) contains 22 keywords, including *accuracy*, *aspiration*, *degulation disorder*, *dysphagia*, *impact*, *infant*, *reliability*, *stroke*, *swallowing*, *symptoms*, *validation*, *vertigo*, and *voice*. Cluster 5 (Purple) contains 21 keywords, including *hearing loss*, *prevalence*, *impairment*, *older adults*, *health*, *dementia*, *depression*, *people*, *risk*, *questionnaire*, *smoking*, *severity*, and *decline*. Cluster 6 (Sky blue) contains 14 keywords, including *COVID-19*, *infection*, *coronavirus*, *disorder*, *dysfunction*, *hyposmia*, *nose*, *smell*, *olfaction*, and *identification*.We created a volcano plot using CiteSpace to visually display how research hotspots have changed over time (Figures 17, 18). We found that *chronic rhinosinusitis*, *SARS-CoV-2*, *vertigo*, *allergic rhinitis*, and *grommets* are current research hotspots.

**Table 7:** High frequency keyword table.

Rank	Keyword	Counts	Rank	Keyword	Counts
1	quality-of-life	91	11	tinnitus	51
2	management	74	12	cochlear implant	49
3	covid-19	71	13	head	45
4	prevalence	68	14	chronic rhinosinusitis	43
5	surgery	66	15	radiotherapy	43
6	children	62	16	impairment	42
7	older-adults	60	17	anosmia	41
8	squamous-cell carcinoma	59	18	impact	41
9	hearing loss	56	19	smell	38
10	survival	51	20	adults	37

### 3.9 Co-cited references and keywords

**Figure 19:**
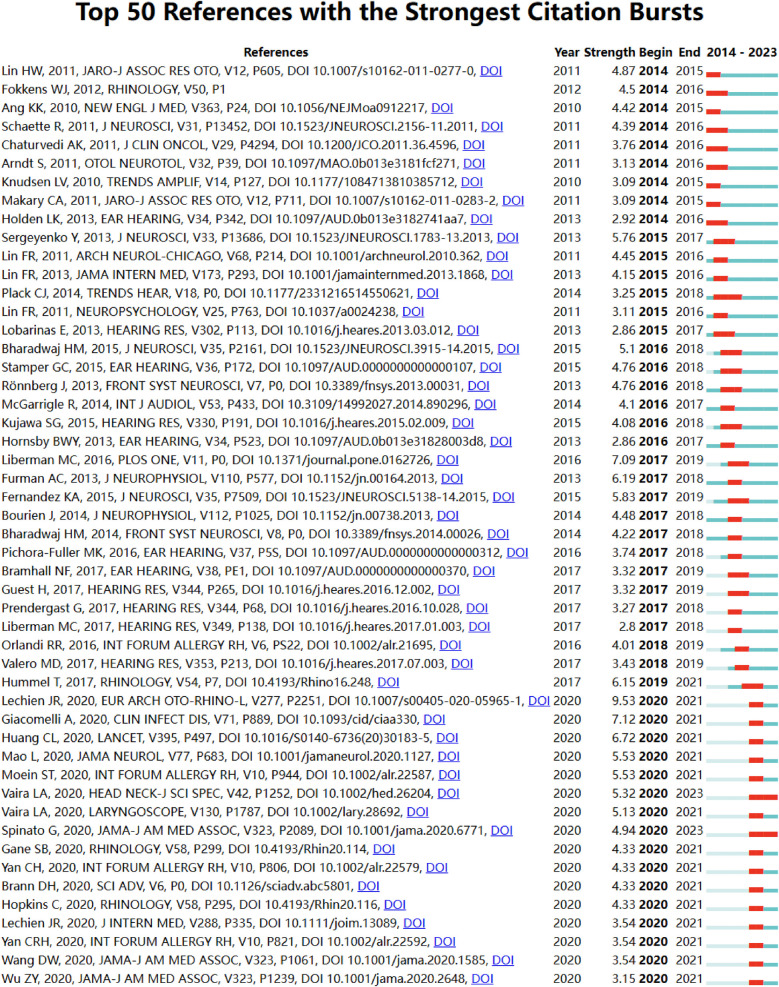
Bursting map of cited literature.

**Figure 20:**
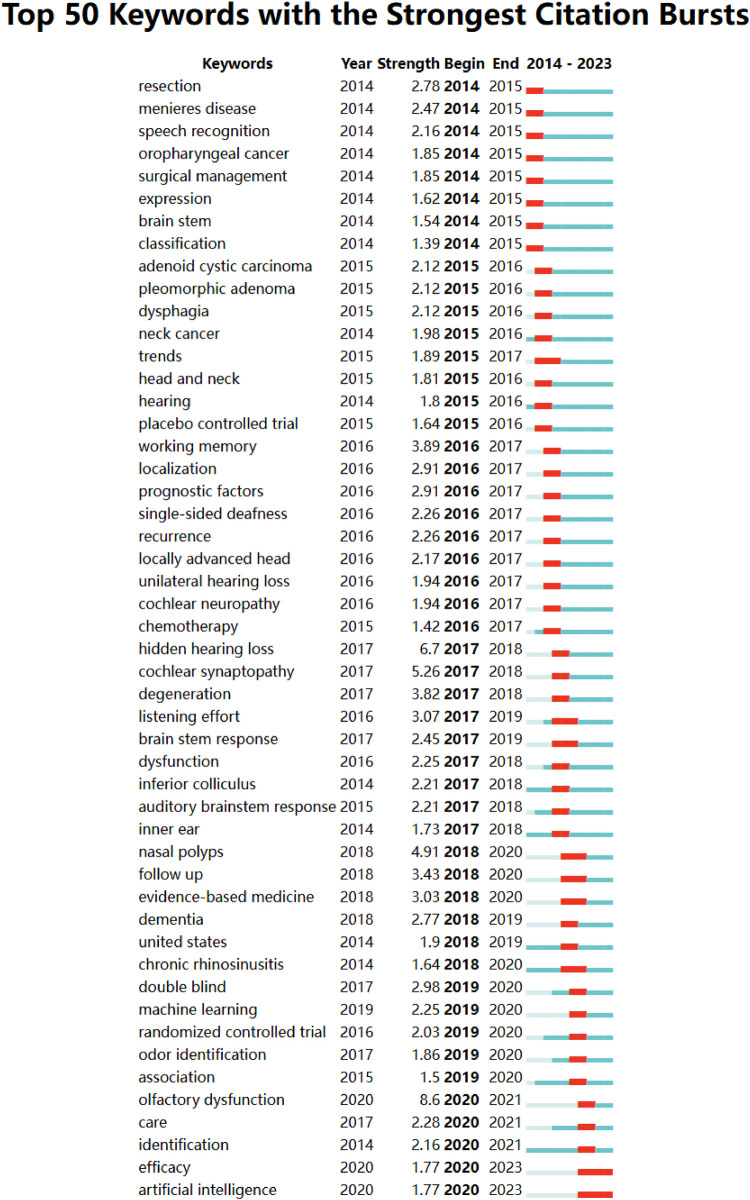
Bursting map of keywords.

Through CiteSpace, we identified the 50 most reliable citation bursts among the 1,000 articles in the field of otolaryngology. After removing irrelevant references, we found that the most frequently cited article was “*Olfactory and gustatory dysfunctions as a clinical presentation of mild-to-moderate forms of the coronavirus disease (COVID-19): a multicenter European study”*, authored by Jerome R. Lechien. This article explored the occurrence of olfactory and gustatory dysfunction in laboratory-confirmed COVID-19 patients. The spread of COVID-19 has sparked increased interest in the study of olfactory and gustatory functions, even among non-otolaryngologists.Of the 50 cited references, 33 were published between 2014 and 2024, indicating that these papers have been frequently cited over the past 10 years. Notably, two of these papers are currently at their peak citation period (Figure 19), suggesting that research related to otolaryngology and artificial intelligence, as well as efficacy-related studies, will continue to be of interest in the future.Among the 466 strongest citation burst keywords in this field, we focused on the 50 keywords with the strongest burst (Figure 20). These keywords represent potential research hotspots in the field of otolaryngology and indicate possible future research directions.

## 4 Discussion

This is an innovative application of quantitative bibliometric methods in otolaryngology research hotspots. It includes the 1,000 most cited research papers in the field of otolaryngology over the past 10 years, retrieved from databases. We analyzed the bibliometric output in the global field of otolaryngology and revealed the main research hotspots and trends between 2014 and 2024. Based on the growth curve, we speculate that an increasing number of non-otolaryngology professionals are showing interest in the field, and the number of publications related to otolaryngology is expected to continue growing.

The United States leads by a large margin in research output in otolaryngology, contributing nearly 60% of the papers. This surpasses the combined total of all other countries. The United States, along with the United Kingdom, Germany, Canada, and Italy, ranks among the top five countries with the highest publication volume. Additionally, the U.S. is also the most cited country, with the citation count of its otolaryngology papers being more than three times that of the second-ranked country. Among the top 10 publishing institutions, 9 are from the United States, highlighting the U.S.'s absolute leadership in the field, reflecting its significant advantage in otolaryngology. Furthermore, the U.S. has the highest centrality score, indicating that it is the most likely partner for collaboration in otolaryngology research worldwide, showing the most prominent cooperation trends. Among the 1,547 institutions included in the study, Harvard University, USA, published the most research. Moreover, the United Kingdom, Italy, Germany, and Canada also had high centrality scores, indicating that they are secondary hubs for otolaryngology research.

Among these 1,000 publications, the most prolific journals in otolaryngology are *Laryngoscope*, *Head and Neck—Journal for the Sciences and Specialties of the Head and Neck*, *Hearing Research*, *Otolaryngology-Head and Neck Surgery*, and *JAMA Otolaryngology-Head & Neck Surgery*. The most co-cited journals are *Laryngoscope* (666 times), followed by *Otolaryngology Head & Neck* (518 times) and *Arch Otolaryngol* (399 times). These publications are world leaders in otolaryngology.

The top 10 co-cited references from 2014 to 2024 indicate that scholars are focusing more on clinical management in otolaryngology. The double-map overlay provides a macroscopic view of the evolution of multidisciplinary research, with the journal's double-map overlay displaying the discipline distribution of academic journals. Otolaryngology research not only involves traditional fields such as medicine, molecular biology, and nursing but is increasingly engaging with psychology, sociology, genetics, and other disciplines. Considering the four main pathways on the map, research in otolaryngology has transitioned from pure clinical studies to interdisciplinary research and the integration of basic and clinical sciences.

Over the past decade, the top five researchers with the most publications include Richard M. Rosenfeld, Zachary M. Soler, Alfio Ferlito, and Thomas Hummel. A clear geographical pattern emerges, with most scholars working in Europe and the United States, primarily in academic centers.

The top 10 high-frequency keywords in co-occurrence cluster analysis indicate that quality of life, hearing loss, and squamous cell carcinoma remain hot topics. Emerging keywords suggest new trends and research frontiers. Frontline areas of otolaryngology have been identified: chronic sinusitis (2020), olfactory disorders (2020), machine learning (2019), odor recognition (2017), artificial intelligence (2018), evidence-based medicine (2018), and chronic rhinosinusitis (2020). Three key trends were identified: nasal polyps (2018–2020), with a citation burst intensity of 4.91, suggesting that the topic suddenly gained significant attention and citations in 2018, becoming a research hotspot. Chronic rhinosinusitis (2014) saw a citation surge between 2018 and 2019, with a sharp increase in citation frequency, likely driven by a major breakthrough or discovery. Machine learning (2019–2020) displayed increased citation activity, showing a sustained interest in the field, with a steady rise in academic impact. This suggests that machine learning is gradually being accepted in otolaryngology, reflecting its continuous development and innovation. A notable event in the application of machine learning to the field of otolaryngology occurred after the outbreak of COVID-19 in 2020, when artificial intelligence was used to diagnose the virus by recognizing cough sounds.

Over the past 10 years, otolaryngology has rapidly become one of the fastest-growing disciplines in medicine, with significant achievements in clinical practice, research, teaching, and interdisciplinary fields. As new theories develop, particularly in interdisciplinary research, numerous new technologies and methods have emerged. The application of big data and artificial intelligence in otolaryngology has injected new vitality into the field, providing a fresh perspective to reassess research directions and development prospects.

In the past decade, advancements in modern otologic microsurgery and otologic imaging have propelled updates in surgical concepts within the field of otolaryngology, particularly by providing more opportunities for hearing restoration based on the complete removal of lesions. Traditional otologic surgery, constrained by surgical approaches and instrumentation, often fails to thoroughly eradicate lesions, leaving blind spots in the procedure. With the continuous development of otologic microsurgery, new surgical concepts have significantly increased the potential for hearing restoration, especially in otoneurosurgery, where modern techniques enable precise localization of lesions. Significant progress has been made in this field, including surgeries on the facial nerve (1), trigeminal nerve (2), vestibular surgery (3), acoustic neuroma resection, and temporal bone-related skull base tumors (4). Simultaneously, research in temporal bone intraoperative navigation technology (5), microsurgical techniques, auricular reconstruction and prosthetics (6), and auditory prosthetics (7) has achieved crucial breakthroughs. Furthermore, the application of artificial intelligence and virtual reality technologies has accelerated post-cochlear implant auditory rehabilitation (8). The use of the Da Vinci robotic system in cochlear implantation surgeries (9) is believed to reduce trauma. The integration of cochlear implants with brain-machine interfaces and artificial intelligence within the framework of transhumanism (10) holds the potential to drive significant breakthroughs in this field. Meanwhile, advances in cochlear hair cell regeneration research (11) have brought hope to patients, with the potential to reverse hearing loss and enhance hearing recovery.

Over the past decade, the continuous development of functional endoscopic nasal surgery and minimally invasive surgical concepts has increasingly focused on the preservation of nasal function, emphasizing the restoration of the patient's natural nasal drainage and minimizing surgical trauma. With advancements in intraoperative navigation and endoscopic technology, otolaryngologists have gained greater confidence in exploring areas once considered taboo. These technologies have enabled the widespread application of endoscopic ophthalmic-related surgeries (such as nasolacrimal duct surgery, optic nerve decompression, and thyroid eye disease decompression), skull base surgeries (such as cerebrospinal fluid leak repair, surgeries for olfactory nerve-related tumors, clival tumors, chordoma surgeries, and pterygopalatine and infratemporal fossa tumor surgeries), and certain intracranial surgeries (such as pituitary adenoma and meningioma surgeries). The surgical scope of otolaryngology continues to expand, with regions such as the petrous apex (12) and craniovertebral junction (13) being increasingly explored by otolaryngologists. The management of the cavernous sinus remains an unavoidable challenge in endoscopic skull base surgery. Its complex anatomy involves multiple important neurovascular structures (such as the optic nerve, oculomotor nerve, and major blood vessels), and intraoperative bleeding from the cavernous sinus often presents significant challenges for surgeons. This is a major challenge that future skull base surgeons will face (14). In the treatment of chronic rhinosinusitis, the use of biologics (15) has opened new directions for precision therapy. Additionally, immunotherapy and targeted therapy for allergic rhinitis and nasal polyps (16) have made significant progress.

Over the past 10 years, significant progress has been made in pharyngeal surgery based on the accumulation of experience. Traditional surgical approaches in sleep surgery, such as nasal airway surgery and Han-UPPP surgery, have significantly improved the treatment outcomes for sleep disorders. Recently emerged surgical methods, such as implantable upper airway support surgery (17), sublingual nerve stimulation implantation (18), robot-assisted pharyngeal surgery (19), and microscopic pharyngeal surgery (20), have proven to be powerful tools in enhancing the quality of life for pharyngeal patients. With the development of voice analysis, laryngeal electromyography, narrowband imaging, and artificial intelligence technologies (21), the diagnosis of voice disorders has become more precise. Advancements in microsurgical techniques and specialized voice microsurgical instruments have provided more accurate means of treating voice disorders, marking a significant step toward precision medicine in voice surgery. Research on vocal cord histopathology has laid a solid theoretical foundation for the treatment of vocal cord diseases. Meanwhile, the development of voice rehabilitation technologies and electronic larynx devices has offered patients who have lost their voice a chance to speak again, providing hope for voice restoration. Research into artificial intelligence-based voice simulation allows electronic larynx devices to closely mimic the patient's original voice, enabling patients who are unable to retain their vocal cords to gradually accept the standard surgical treatment of complete vocal cord removal.

Over the past 10 years, head and neck surgery has made significant advancements, evolving from traditional radical surgeries to a focus on improving long-term patient outcomes and implementing comprehensive treatment strategies. The development of flap and functional reconstruction techniques has provided head and neck surgeons with more robust tools, enabling the treatment of lesions that were previously difficult to repair. Virtual reality and 3D printing technologies (22) have brought new breakthroughs to flap design, further advancing this field. With the increasing emphasis on functional preservation, head and neck surgery now not only aims to save patients' lives but also considers their long-term quality of life, thus achieving the maximum comprehensive benefit. The continued progress in radiotherapy and chemotherapy has further extended the survival of head and neck cancer patients, and the concept of comprehensive treatment has become widely accepted, moving beyond the reliance on traditional radical resection. In recent years, immunotherapy for head and neck tumors (23) has become a hot topic, with its personalized and precise treatment characteristics believed to fundamentally alter the existing treatment landscape. Furthermore, advancements in early diagnostic techniques and the research on molecular circulating tumor markers (24) have also achieved notable successes. Robotic-assisted head and neck surgery has enhanced the safety of procedures (25), while ultrasound-guided treatment of neck tumors (26) and radioactive particle implantation (27) have provided new treatment options for patients. The application of immune checkpoint inhibitors and the development of targeted therapies (28) offer new treatment hopes for patients with recurrence or those unable to tolerate surgery. The progress in facial nerve preservation and repair techniques (29) has provided patients with innovative treatment solutions.

Currently, otolaryngology is facing numerous development opportunities, particularly in the following research hotspots: In otology, current research focuses on the integration of cochlear implants with artificial intelligence technology, aiming to achieve more precise sound processing, especially in improving speech recognition in noisy environments; hearing restoration and neuroplasticity research post-cochlear implant; multidisciplinary comprehensive treatment for tinnitus; and gene therapy research for hearing loss. In rhinology, the main research directions include: immunological mechanisms of chronic rhinosinusitis and nasal polyps; discovery of biomarkers and precision treatment for chronic rhinosinusitis and nasal polyps; and the relationship between the nasal microbiome and nasal diseases. In voice and head and neck disease research, key areas include: targeted therapy and immunotherapy for head and neck tumors; vocal cord repair and regeneration techniques; tissue engineering and head and neck reconstruction research; and the application of artificial intelligence and machine learning in otolaryngology, particularly in automatic image recognition, treatment plan automation, early diagnosis, and assisted surgical planning. Additionally, robot-assisted surgery in the head and neck region has emerged as a new research direction.The interdisciplinary collaboration of finite element analysis and artificial intelligence assistance not only plays a huge advantage in traditional disciplines such as science and engineering (30, 31), but also plays an increasingly important role in improving ear, nose, and throat diagnostic methods and updating treatment plans.

In conclusion, this study, through the analysis of the 1,000 most influential papers in otolaryngology over the past 10 years, visualizes the research dynamics and development trajectory of the otolaryngology discipline. Our analysis results show that the interdisciplinary development of otolaryngology, especially in genetics, artificial intelligence, and precision medicine, has become one of the driving forces in the advancement of otolaryngology research. Future trends suggest that precision medicine and the integration of AI with clinical practice will play an increasingly important role. Research directions should focus on translational medicine and the development of high-level interdisciplinary teams. Through continuous development of innovative technologies and deepening clinical research, otolaryngology is expected to usher in new opportunities.

## Data Availability

The original contributions presented in the study are included in the article/Supplementary Material, further inquiries can be directed to the corresponding author.
