# Correction: Bibliometric analysis of the top 1000 most-cited articles in otolaryngology over the past decade: global research trends and hotspots

**DOI:** 10.3389/fsurg.2025.1769697

**Published:** 2026-01-02

**Authors:** Zhipeng Wang, Guodong Yu

**Affiliations:** 1 Department of Otolaryngology, Affiliated Hospital of Guizhou Medical University, Guiyang, China; 2 School of Clinical Medicine, Guizhou Medical University, Guiyang, China; 3 Guizhou Provincial People’s Hospital Hearing Center, Guiyang, China

**Keywords:** bibliometric analysis, otolaryngology, review, discipline development, ENT

Affiliation 3 “Guizhou Provincial People's Hospital Hearing Center, Guiyang, China” was omitted for author Zhipeng Wang. This affiliation has now been added.

The original version of this article has been updated.

